# Plant Growth-Promoting Nitrogen-Fixing Enterobacteria Are in Association with Sugarcane Plants Growing in Guangxi, China

**DOI:** 10.1264/jsme2.ME11275

**Published:** 2012-04-18

**Authors:** Li Lin, Zhengyi Li, Chunjin Hu, Xincheng Zhang, Siping Chang, Litao Yang, Yangrui Li, Qianli An

**Affiliations:** 1 State Key Laboratory for Conservation and Utilization of Subtropical Agro-bioresources, Guangxi University, Daxuedong Road 100, Nanning 530004, China; 2 Guangxi Academy of Agricultural Sciences, Daxuedong Road 174, Nanning 530007, China; 3 Sugarcane Research Center, Chinese Academy of Agricultural Sciences, Daxuedong Road 174, Nanning 530007, China; 4 Institute of Biotechnology, Zhejiang University, Zijinggang Campus, Hangzhou 310058, China

**Keywords:** biological nitrogen fixation, enterobacteria, *nifH*, plant growth-promoting bacteria, sugarcane

## Abstract

The current nitrogen fertilization for sugarcane production in Guangxi, the major sugarcane-producing area in China, is very high. We aim to reduce nitrogen fertilization and improve sugarcane production in Guangxi with the help of indigenous sugarcane-associated nitrogen-fixing bacteria. We initially obtained 196 fast-growing bacterial isolates associated with the main sugarcane cultivar ROC22 plants in fields using a nitrogen-deficient minimal medium and screened out 43 nitrogen-fixing isolates. Analysis of 16S rRNA gene sequences revealed that 42 of the 43 nitrogen-fixing isolates were affiliated with the genera *Enterobacter* and *Klebsiella*. Most of the nitrogen-fixing enterobacteria possessed two other plant growth-promoting activities of IAA production, siderophore production and phosphate solubilization. Two *Enterobacter* spp. strains of NN145S and NN143E isolated from rhizosphere soil and surface-sterilized roots, respectively, of the same ROC22 plant were used to inoculate micropropagated sugarcane plantlets. Both strains increased the biomass and nitrogen content of the sugarcane seedlings grown with nitrogen fertilization equivalent to 180 kg urea ha^−1^, the recommended nitrogen fertilization for ROC22 cane crops at the seedling stage. ^15^N isotope dilution assays demonstrated that biological nitrogen fixation contributed to plant growth promotion. These results suggested that indigenous nitrogen-fixing enterobacteria have the potential to fix N_2_ associated with sugarcane plants grown in fields in Guangxi and to improve sugarcane production.

Guangxi is the major sugarcane- and sugar-producing area in China and produces about 60% of China’s sugarcane and sugar. The present sugarcane mean yields are between 70 and 80 Mg ha^−1^. The cost of sugarcane production in Guangxi is much higher than in Brazil. One of the major factors of the high cost is high N-fertilization. Over 60% of the sugarcane fields are applied with urea at over 600 kg ha^−1^ yr^−1^ (32). In Brazil, the present sugarcane mean yields are also between 70 and 80 Mg ha^−1^, but N-fertilizer mean applications are between 60 and 70 kg N ha^−1^ yr^−1^ (50). ^15^N isotope assays have demonstrated that some Brazilian sugarcane varieties are able to obtain considerable nitrogen from biological nitrogen fixation (BNF; 25, 49, 50). A number of nitrogen-fixing bacteria have been isolated from the rhizosphere and interior of sugarcane plants, and have shown potential to fix N_2_ associated with sugarcane plants (5, 13, 35, 43).

BNF may help farmers to maintain sugarcane yields under reduced N-fertilization and develop environmentally benign sugarcane production in Guangxi. At present, little is known about the diversity and predominant population of nitrogen-fixing bacteria associated with the sugarcane plants growing in Guangxi. Recently, some nitrogen-fixing bacteria have been isolated from sugarcane plants grown in Guangxi (26, 28, 44, 52, 53) using NFb, JNFb and LGI-P media that were respectively used to isolate *Azospirillum* (10), *Herbaspirillum* (6) and *Gluconacetobacter diazotrophicus* (8); however, nitrogen-fixing bacteria belonging to the genera *Azospirillum*, *Herbaspirillum*, and *Gluconacetobacter*, which are predominantly associated with sugarcane plants in Brazil, have not been isolated. The diversity of nitrogen-fixing bacteria associated with sugarcane plants grown with high N-fertilization in Guangxi may be different from in Brazil.

The ROC22 cultivar is the main sugarcane cultivar, growing in over 60% of sugarcane-planting areas in Guangxi. It is sensitive to low nitrogen stress and requires at least 150 kg ha^−1^ urea fertilization at the seedling stage for tillering and elongation of the plant cane crops (23). The recommended dose of urea fertilization for plant cane crops at the seedling stage is 180 kg ha^−1^, 30% of urea fertilization for a season (46). Recent studies have shown that nitrogen-fixing bacterial strains isolated from other sugarcane cultivars are able to provide nitrogen to micropropagated ROC22 sugarcane seedlings via BNF and promote sugarcane growth (26, 29); however, neither indigenous nitrogen-fixing bacteria associated with ROC22 sugarcane plants nor their associative BNF under recommended N-fertilization have been investigated.

Here, we attempted to isolate a large number of nitrogen-fixing bacteria associated with ROC22 sugarcane plants, investigate their diversity and predominant affiliation, and evaluate their potential for plant-growth promotion and associative BNF under the recommended N-fertilization. We initially obtained 196 fast-growing isolates from rhizosphere soil and roots of ROC22 sugarcane plants grown in 14 production areas. Nitrogen-fixing isolates were screened using the acetylene reduction assay (ARA) and PCR amplification of the *nifH* gene encoding the iron protein of nitrogenase (55). We found that enterobacteria were predominant among the obtained nitrogen-fixing bacteria by analyzing their 16S rRNA gene (*rrs*) sequences. We further screened their plant growth-promoting activities, including the production of indole acetic acids (IAA) and siderophores, phosphate solubilization and ACC (1-aminocyclopropane-1-carboxylic acid) deamination. Finally, we chose two *Enterobacter* spp. strains isolated from the same ROC22 plant to inoculate micropropagated ROC22 sugarcane seedlings and investigate their plant growth-promoting and associative BNF activities under the recommended N-fertilization for ROC22 crops using the ^15^N isotope dilution technique.

## Materials and Methods

### Bacterial isolation

Root samples were taken from ROC22 sugarcane plants grown for five to eight months in the fields in 14 production areas (Table 1). Root systems of six sugarcane plants in each production area were dug out; bulk soil loosely adhering to the roots was shaken off; rhizosphere soil tightly adhering to the roots was suspended in autoclaved distilled water; roots were washed in sequence once by autoclaved distilled water, 70% (v/v) ethanol for 30 s, 0.1% (w/v) HgCl_2_ for 1 min and 70% (v/v) ethanol for 30 s, five times by autoclaved distilled water, and ground with autoclaved quartz sand and phosphate-buffered saline (40) with a mortar and pestle. Soil suspensions and root homogenates were 10-fold serially diluted using autoclaved distilled water. One hundred microliters of each suspension were spread on modified nitrogen-deficient Ashby’s agar medium (per liter contains 10 g sucrose, 0.2 g NaCl, 0.2 g KH_2_PO_4_, 0.2 g MgSO_4_·7H_2_O, 0.1 g CaSO_4_·2H_2_O, 5 g CaCO_3_, 15 g agar, pH 7.0) (3). After incubation at 30°C for 3–5 d, colonies with distinguished morphology were purified three times by streaking on Ashby’s agar. Purified isolates were maintained on Luria-Bertani (LB) agar (40). Their liquid LB cultures were stored with 15% (v/v) glycerol at −70°C.

### ARA

One milliliter of each isolate grown overnight in liquid LB medium was harvested, washed twice, and suspended in 10 mL of liquid Ashby’s medium in a 60-mL Erlenmeyer flask. After static incubation at 28°C for 24 h, the flask was sealed with a rubber stopper and then 5 mL (10%) gas volume in the flask was replaced with acetylene. After incubation for another 24 h, ethylene was detected with a Shimadzu GC-9A gas chromatograph (Shimadzu, Kyoto, Japan) equipped with a flame ionization detector and a column filled with GDX-502 (Borui Jianhe Chromatography Technology, Tianjin, China) (53). To ARA-negative in initial ARA screening but *nifH*-positive isolates of LA16S, DX194E and LC89E, each isolate was suspended in modified liquid Ashby’s medium supplemented with 0.05% (w/v) yeast extract in flasks at 28°C; ARA was performed every 2 h on bacterial cultures during a 24 h growth period.

### Colony PCR

A colony approximately 1 mm in diameter grown on LB agar was picked up with an autoclaved 10-μL pipette tip and transferred to 10 μL sterilized Millipore water in a PCR tube. The bacterial suspension was heated in a P7021TP-6 microwave oven (Galanz, Foshan, China) at full power for 3 min. After centrifugation, 1 μL of the bacterial lysate was used as the template for PCR. The *G. diazotrophicus* strain PAL5 isolated from sugarcane plants (13) and the *Escherichia coli* strain DH5α were respectively used as positive and negative controls for *nifH* amplification.

Amplification of partial *nifH* sequences was performed with the degenerate Z-primers (55). Amplification of near full-length *rrs* sequences was performed with universal 27F/1492R primers (22). The primers were synthesized by Sangon (Sangon Biotech, Shanghai, China) and Takara (Takara Biotechnology, Dalian, China). The primers synthesized by Sangon were purified by ULTRAPAGE and determined by mass spectrometry. The primers synthesized by Takara were purified by HPLC. The Z-primers were used at 2 μM. The 27F/1492R primers were used at 0.25 μM. Ready-to-use 2×concentrated PCR masters (0.1 U μL^−1^ *Taq* DNA polymerase, 0.2 mM dNTP, 3 mM MgCl_2_, 2×PCR buffer) produced by Sangon and Tiangen (Tiangen Biotech, Beijing, China) were used for reactions. PCR amplification was performed in a PTC-200 DNA Engine thermal cycler and an S1000 thermal cycler (Bio-Rad Laboratories, Hercules, CA, USA). A touchdown PCR strategy was used for *nifH* amplification to improve amplification specificity. In the 20 touchdown cycles, the annealing temperature was decreased by 0.5°C every cycle from 67 to 57°C. Fifteen additional cycles were performed at the annealing temperature of 57°C. PCR products were electrophoresed on agarose gels, stained with ethidium bromide, visualized and recorded with a JS-680B Gel Documentation and Analysis System (Shanghai Peiqing Science and Technology, Shanghai, China), and compared with molecular weight markers (Takara).

### Cloning and sequencing of *nifH* and *rrs* fragments

The expected amplicons were excised from agarose gels, purified by a TIANgel Midi Purification Kit (Tiangen), cloned into the pMD18-T vector (Takara) and screened as described by the manufacturers. Three to six clones containing inserts of the correct size for each amplicon were chosen for sequencing (Invitrogen, Carlsbad, CA, USA).

### Sequence analysis

The sequence segments corresponding to Z-primers were removed from the amplified *nifH* sequences. The partial *nifH* sequences were translated using the MEGA 4.0 program (45). The deduced amino acid sequences were aligned using the MUSCLE program (11) implemented in the MEGA 4.0 program. The *rrs* sequences were screened with the Mallard program (4) and BLASTed (1). The *rrs* sequences of enterobacteria were aligned with those from the type strains of the species belonged to the genera *Enterobacter* and *Klebsiella* in the List of Prokaryotic names with Standing in Nomenclature (http://www.bacterio.cict.fr/m/microbacterium.html) using the MUSCLE program. Phylogenetic analysis was performed using the PhyML 3.0 program based on the maximum-likelihood principle (16). The best-fit TIM1+G model of nucleotide substitution was estimated using the jModelTest program based on the Akaike information criterion (38). The branch support measurement was assessed by a nonparametric, Shimodaira-Hasegawa-like test implemented in the PhyML 3.0 program.

### Determination of IAA production, siderophore production, phosphate solubilization, and ACC deaminase activity

IAA production from a 3-d culture in DF medium (36) supplemented with 0.02% (w/v) L-tryptophan was determined by a microplate colorimetric assay (41). Siderophore production was determined by the Chrome Azurol S plate assay (42). Mineral phosphate solubilization activity was determined using Pikovskaya’s agar medium containing 0.5% (w/v) Ca_3_(PO_4_)_2_ (37). Bacterial utilization of ACC was screened using a colorimetric assay of ACC based on the ninhydrin reaction (24); bacterial ACC deaminase activity was measured as described by Penrose and Glick (2003) (36).

### Inoculation and acclimatization of micropropagated sugarcane plantlets

Sugarcane micropropagated plantlets were developed from stem apical meristems of cultivar ROC22 (51). Two nitrogen-fixing *Enterobacter* spp. strains of NN145S and NN143E isolated from rhizosphere soil and surface-sterilized roots, respectively, of the same ROC22 plant were used to inoculate micropropagated plantlets. Both strains were also able to produce siderophores and solubilize Ca_3_(PO_4_)_2_. Inoculation and acclimatization of micropropagated plantlets were performed as described by Lin *et al.* (26). Rooted plantlets were co-cultured with bacteria in liquid one-tenth MS medium (sucrose and basal salt mixture) (39). Initial density of the bacteria was approximately 2.0×10^5^ cells per milliliter medium. Plantlets without inoculation were prepared as the control. Seven days after inoculation, plantlets were transferred to autoclaved sands and acclimatized for 14 d (26).

### Counting of NN145S and NN143E cells colonizing sugarcane plantlets

Seven days after inoculation, plantlets were briefly washed with autoclaved distilled water, dried with autoclaved tissue paper, weighed, and ground with autoclaved quartz sand and phosphate-buffered saline in a mortar and pestle. The homogenates were serially diluted and spread on Ashby’s agar. Bacterial colonies were counted after incubation at 30°C for 3 d.

### ^15^N isotope dilution assay

Sand and perlite were mixed 1:1 (v:v), autoclaved twice at 121°C for 2 h, and cooled for 1 d at room temperature after each autoclave. The soil mixtures were added to 10 mg (NH_4_)_2_SO_4_ (10.11 atom % ^15^N excess) per kilogram wet weight of soil mixture (kg^−1^ soil) and mixed thoroughly twice daily for 2 weeks prior to planting to ensure uniform distribution of ^15^N isotope (19). Two and a half kilograms of ^15^N-labeled soil mixtures filled each autoclaved 2.5-L plastic pot. Each pot was then planted with three acclimatized micropropagated sugarcane seedlings. Six seedlings for each treatment were fertilized by adding 200 mL of one-tenth MS basal salt mixture to each pot 5 d after transplant. As a result, 8.82 mg N kg^−1^ soil including ^15^N isotope was added. Oliveira *et al.* (35) estimated that 10 mg (NH_4_)_2_SO_4_ (2.1 mg N) kg^−1^ soil is equivalent to 20 kg N ha^−1^. The N-fertilization of 8.82 mg N kg^−1^ soil is thus equivalent to 84 kg N ha^−1^ or 180 kg urea ha^−1^. The seedlings were grown under a 14-h light period with approximately 100 μmol m^−2^ s^−1^ photon flux density at 26±2°C, and watered as needed with autoclaved distilled water. Fifty-five days after transplant, plant roots were washed by distilled water to remove the attached soil mixtures. Roots and shoots were separated, freeze-dried, weighed, and ground. Ten milligrams of ground root and shoot materials were respectively analyzed for ^15^N isotope content by a Delta Plus AD EA-IRMS Mass Spectrometer (Thermo Fisher Scientific, Waltham, MA, USA). The contribution of nitrogen derived from air (Ndfa) was estimated by the equation: %Ndfa=100×(1−[atom % ^15^N excess inoculated plant/atom % ^15^N excess control plant]) (19). Data plotting and statistical T test were carried out using Microsoft Excel 2007. The confidence intervals were 95% (α=0.05).

### Nucleotide sequence accession numbers

The partial *nifH*, *anfH* (encoding the iron protein of Fe-only alternative nitrogenase; 20) and *rrs* sequences were deposited in GenBank under accession numbers HQ204222 to HQ204264, HQ204265 to HQ204276, and HQ204277 to HQ204319, respectively (Table S1).

## Results

### Bacterial isolation and ARA screening

Bacterial colonies grown well on nitrogen-deficient Ashby’s agar generally appeared from 10-fold diluted soil suspensions and root homogenates and were selected for purification. One hundred ninety-six fast-growing isolates were obtained. Acetylene reduction activity in liquid nitrogen-deficient Ashby’s medium was repeatedly detected from 18 isolates obtained from rhizosphere soil and 22 isolates obtained from surface-sterilized roots.

### PCR amplification and sequencing confirmation of partial *nifH* genes

PCR amplification of *nifH* (*nifH* PCR) based on Z-primers amplified approximately 360 bp fragments from all 40 ARA-positive isolates (Fig. S1A). Cloning and sequencing of the amplicons showed that all 40 ARA-positive isolates contained sequences that were the closest match to *nifH* (Table S1). In addition, sequences matching *anfH* encoding the iron protein of Fe-only alternative nitrogenase (20) were obtained from 12 isolates (Table S1). The conserved residues of C86, C98, R101, D126, D130, and C133 (*Azotobacter vinelandii* OP NifH numbering; GenBank accession number AAA64709) for nitrogenase iron protein were present in all the obtained partial NifH and AnfH sequences (data not shown).

### Application of Z-primer-based colony *nifH* PCR

In the initial screening of the 196 bacterial isolates, ARA did not detect nitrogenase activity from 156 isolates; however, ARA may miss true nitrogen-fixing bacteria whose nitrogenase activities would be induced under more favorable growth conditions or at more active growth stages. To verify this assumption, we used Z-primer-based colony *nifH* PCR for the 156 ARA-negative isolates. Positive amplification was obtained from three isolates of LA16S, DX194E, and LC89E (Fig. S1B). Subsequent cloning and sequencing confirmed that the three isolates contained *nifH* (Table S1). We further performed ARA on the three isolates grown in liquid Ashby’s medium supplemented with 0.05% (w/v) yeast extract. Bacterial growth was promoted by yeast extract and reached the exponential phase after 4 h. Weak acetylene reduction activity was detected from the cultures of three isolates grown for 8–16 h (data not shown); therefore, Z-primer-based colony *nifH* PCR is able to rapidly screen *nifH*-positive isolates from a large number of bacterial isolates and largely reduce the number of isolates for further ARA determination.

### IAA production, siderophore production, phosphate solubilization and ACC deaminase activity

Among the 43 nitrogen-fixing bacterial isolates, 42 isolates produced siderophores; 34 isolates including 19 isolates obtained from rhizosphere soil and 15 isolates obtained from roots were able to dissolve Ca_3_(PO_4_)_2_; 27 isolates including 13 isolates obtained from rhizosphere soil and 14 isolates obtained from roots produced IAA; one isolate CZ152S displayed ACC deaminase activity (24); 19 isolates showed two of the four tested plant growth-promoting activities; 21 isolates showed three of the four activities (Table 1).

### Analysis of *rrs* sequences

**Fig. 1:**
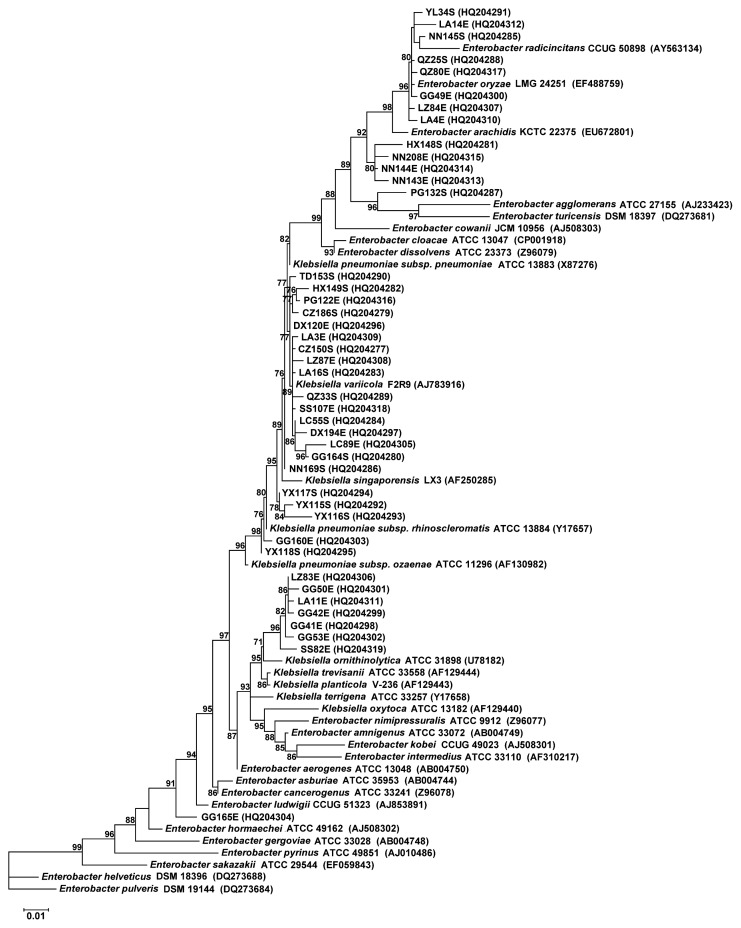
Phylogenetic tree based on 16S rRNA gene sequences for nitrogen-fixing enterobacteria associated with sugarcane cultivar ROC22 and the type strains of the species belonging to the genera *Enterobacter* and *Klebsiella*. Accession numbers of the 16S rRNA genes in the GenBank database are given in parentheses. The tree was generated by the maximum-likelihood principle using the PhyML 3.0 program. The TIM1+G model was used. Branch support measure was assessed by a Shimodaira-Hasegawa-like test and values >50 are indicated at the nodes. Bar, 0.01 nucleotide substitutions per site.

Approximately 1,500 bp of *rrs* sequences for all 43 nitrogen-fixing isolates were obtained by PCR amplification and confirmed by subsequent cloning and sequencing. Sequence BLASTing suggested that 28 isolates belonged to the genus *Klebsiella*, 14 isolates belonged to the genus *Enterobacter*, and isolate CZ152S belonged to the genus *Burkholderia* (Table 1 and S1). The *rrs* sequence of CZ152S showed the highest sequence similarity to those of *B. cepacia* strain LMG 12614 (99.8%) and *B. cenocepacia* strain J2315 (99.8%). The phylogenetic tree of the *rrs* sequences of the 28 *Klebsiella* spp. isolates, 14 *Enterobacter* isolates, and the type strains of the *Klebsiella* and *Enterobacter* species are shown in Fig. 1.

### Inoculation effects on micropropagated sugarcane seedlings

**Fig. 2:**
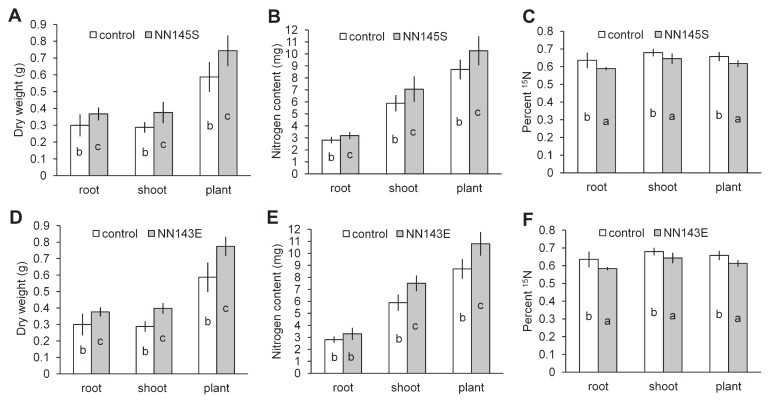
Inoculation effects of *Enterobacter* sp. NN145S (A, B, C) and NN143E (D, E, F) on micropropagated sugarcane ROC22 seedlings. (A, D) dry weight, (B, E) nitrogen content, and (C, F) percent ^15^N of roots, shoots, and whole seedlings are presented for comparison of inoculated seedlings with uninoculated controls. The columns represent the mean of the data for each treatment. Bars represent the standard error. Different letters within the columns indicate significant differences between the treatments at the 95% confidence level.

Seven days after inoculation, approximately 6.5×10^7^ NN145S cells and 8.0×10^7^ NN143E cells per gram of the micropropagated sugarcane plantlets were counted, whereas no bacterial colonies were formed from uninoculated controls. After 14 d acclimatization without N-fertilization and 55 d growth with 8.82 mg N kg^−1^ soil including ^15^N isotope, inoculated sugarcane seedlings showed higher dry weights and nitrogen contents than those of uninoculated controls (Fig. 2). Inoculation of strain NN145S increased the dry weights of roots, shoots, and whole seedlings at 22.7%, 30.6%, and 26.7%, respectively (Fig. 2A), and increased nitrogen contents of roots, shoots, and whole seedlings at 13.7%, 20.0%, and 17.9%, respectively (Fig. 2B); all the increases were statistically significant at the 95% confidence level (Fig. 2A and B). Inoculation of strain NN143E increased dry weights of roots, shoots, and whole seedlings at 25.6%, 38.4%, and 31.9%, respectively (Fig. 2D), and increased nitrogen contents of roots, shoots, and whole seedlings at 17.2%, 27.4%, and 24.0%, respectively (Fig. 2E); the increases were statistically significant at the 95% confidence level, except that the increase of the root nitrogen content was significant at the 90% confidence level (Fig. 2D and E). Moreover, the ^15^N isotope concentrations of the inoculated plants were significantly lower than those of the uninoculated controls (Fig. 2C and F) as a result of dilution by N_2_ fixation. Seedlings inoculated with NN145S received 7.3%, 5.0%, and 6.1% nitrogen in roots, shoot, and whole plants from N_2_, respectively; seedlings inoculated with NN143E received 8.3%, 5.3%, and 6.7% nitrogen in roots, shoot, and whole plants from N_2_, respectively. The dry weight (0.77 g plant^−1^), nitrogen content (10.79 mg plant^−1^), and %Ndfa (6.7) of sugarcane seedlings inoculated with NN143E were respectively higher than those (0.74 g plant^−1^, 10.26 mg plant^−1^, and 6.1) of seedlings inoculated with NN145S but were not statistically significant.

## Discussion

This initial large-scale isolation of nitrogen-fixing bacteria from root samples of the main sugarcane cultivar ROC22 grown in Guangxi obtained a limited number of nitrogen-fixing isolates that showed limited diversity and predominantly belonged to the genera *Klebsiella* and *Enterobacter*. Previous studies showed that high levels of N-fertilization reduced the bacterial cell number (12, 34) and genetic diversity (7) of *G. diazotrophicus* in sugarcane plants. The high levels of N-fertilization in the sugarcane fields in Guangxi may act as a selective factor to reduce the population and diversity of nitrogen-fixing bacteria.

Enterobacteria are seemingly predominant in the fast-growing culturable nitrogen-fixing bacteria associated with ROC22 sugarcane plants growing in Guangxi. On the one hand, the selective nitrogen-deficient Ashby’s medium used in the isolation procedure may lead to the predominant isolation of enterobacteria. On the other hand, nitrogen-fixing enterobacteria have been isolated from sugarcane plants cultivated in Guangxi using NFb and JNFb media (28, 44, 53) and also isolated from sugarcane plants cultivated in other countries (15, 27, 31, 33, 48). Mehnaz *et al.* (31) found that enterobacteria were predominant among the nitrogen-fixing bacteria isolated from sugarcane plants in Pakistan. Taulé *et al.* (48) found that enterobacteria formed a major group among the endophytic nitrogen-fixing bacteria isolated from sugarcane plants in Uruguay. Magnani *et al.* (30) showed that enterobacteria, including three nitrogen-fixing isolates, comprised a major group of endophytic bacteria isolated from Brazilian sugarcane using potato agar medium. Moreover, the detection of *nifH* sequences in sugarcane plants grown in Japan without culture showed that *nifH* sequences homologous to those of enterobacteria were predominant (2). The frequent association between nitrogen-fixing enterobacteria and sugarcane crops may develop from the long use of organic manures in agriculture (21).

Recent studies have shown that nitrogen-fixing enterobacteria can fix N_2_ associated with sugarcane plants and promote sugarcane growth. Luo *et al.* (29) showed that a *Klebsiella* sp. strain fixed N_2_ and increased nitrogen content of the ROC22 seedlings under gnotobiotic conditions. Wu *et al.* (52) showed that a nitrogen-fixing *Pantoea* sp. strain promoted the growth of sugarcane plants grown in non-sterile sand. Govindarajan *et al.* (15) showed that a nitrogen-fixing *Klebsiella* sp. strain increased the biomass and nitrogen content of sugarcane plants grown in non-sterile soil. Mirza *et al.* (33) showed that a *Klebsiella* sp. isolate SC20 (identified on the basis of its *rrs* sequence [GenBank accession number AJ278447]) fixed N_2_ and increased the nitrogen content of sugarcane plants under gnotobiotic conditions. Here, we showed that two *Enterobacter* spp. strains provided nitrogen to sugarcane plants via BNF and promoted sugarcane growth under gnotobiotic conditions and relatively high N-fertilization (equivalent to 180 kg urea ha^−1^) recommended for sugarcane plant crops at the seedling stage in Guangxi. To our knowledge, this is the first report that indigenous nitrogen-fixing *Enterobacter* spp. strains can fix N_2_ associated with the hosts of sugarcane plants and promote their growth.

Using the method of introducing endophytic bacteria into the rooted micropropagated sugarcane plantlets established by Reis *et al.* (39), both the rhizosphere soil isolate NN145S and the root isolate NN143E obtained from the same ROC22 sugarcane plant were able to heavily colonize the ROC22 seedlings at respective densities of 6.5×10^7^ and 8.0×10^7^ cells per gram fresh weight. It would be interesting to study their colonization patterns, interactions and coordinated BNF contributions after coinoculation into the host ROC22 plant.

ARA is a sensitive method to detect nitrogenase activity of microbial cultures and has been widely used to identify nitrogen-fixing isolates (17). It is also known that bacterial nitrogenase activities vary with the media, culture conditions, and growth stages; therefore, using ARA to screen diazotrophs from a large number of isolates may miss true diazotrophs in the silent state of N_2_ fixation and is rather time-consuming. Here, we have shown that colony *nifH* PCR can circumvent the disadvantages of ARA and rapidly screen nitrogen-fixing bacteria from a large number of isolates. Firstly, *nifH* PCR amplification based on Z-primers is sensitive and specific for the identification of ARA-positive isolates belonging to broad taxonomic classes (9, 18, 47). Demba Diallo *et al.* (9) have revealed that Z-primers have the highest rate of match to *nifH* sequences in the databases among the widely used universal *nifH* primers. Moreover, Z-primer-based PCR can amplify the corresponding fragments of *nifH*, *vnfH*, and *anfH* (9, this study). Secondly, colony *nifH* PCR skipping DNA extraction is easy to perform, rapid, reproducible, and not related to bacterial growth stages. Commercially available reagents, such as the ready-to-use 2×concentrated PCR masters, can facilitate PCR manipulation. In a modern microbiology laboratory, a thermal cycler for PCR is usually more available than a gas chromatograph for ARA. Using colony *nifH* PCR for the initial screening can largely reduce the number of isolates for further ARA determination; however, great care should be taken regarding the contamination of primers and other PCR reagents by trace levels of *nifH* (14, 54).

In conclusion, colony *nifH* PCR followed by ARA determination of *nifH*-positive isolates enables highly efficient workflow to screen and identify diazotrophs. The initial large-scale isolation of nitrogen-fixing bacteria from ROC22 sugarcane root samples obtained predominantly nitrogen-fixing enterobacteria that possessed multiple plant growth-promoting activities of IAA production, siderophore production, or phosphate solubilization. The initial inoculation of two *Enterobacter* spp. strains showed the potential for nitrogen-fixing enterobacteria to provide nitrogen via BNF to sugarcane cultivars requiring a high level of N-fertilization and increase sugarcane production in fields with relative high N-fertilization in Guangxi.

## Supplementary Material



## Figures and Tables

**Table 1:** Nitrogen-fixing bacterial isolates associated with sugarcane cultivar ROC22 grown in Guangxi

Isolates	Isolation site	Isolation source	Genus affiliation	IAA production	Siderophore production	Phosphate solubilization	ACC deaminase
CZ150S	Chongzuo	soil	*Klebsiella*	+	+	+	−
CZ152S	Chongzuo	soil	*Burkholderia*	−	+	+	+
CZ186S	Chongzuo	soil	*Klebsiella*	+	+	+	−
GG164S	Guigang	soil	*Klebsiella*	+	+	+	−
HX148S	Hengxian	soil	*Enterobacter*	−	+	+	−
HX149S	Hengxian	soil	*Klebsiella*	+	+	+	−
LA16S	Longan	soil	*Klebsiella*	+	+	+	−
LC55S	Liuchen	soil	*Klebsiella*	+	+	+	−
NN145S	Nanning	soil	*Enterobacter*	−	+	+	−
NN169S	Nanning	soil	*Klebsiella*	+	+	+	−
PG132S	Pingguo	soil	*Enterobacter*	+	+	+	−
QZ25S	Qinzhou	soil	*Enterobacter*	−	+	+	−
QZ33S	Qingzhou	soil	*Klebsiella*	−	+	+	−
TD153S	Tiandong	soil	*Klebsiella*	+	+	+	−
YL34S	Yulin	soil	*Enterobacter*	−	+	+	−
YX115S	Yangxi	soil	*Klebsiella*	+	+	+	−
YX116S	Yangxi	soil	*Klebsiella*	+	+	+	−
YX117S	Yangxi	soil	*Klebsiella*	+	+	+	−
YX118S	Yangxi	soil	*Klebsiella*	+	+	+	−
DX120E	Daxin	root	*Klebsiella*	+	+	+	−
DX194E	Daxin	root	*Klebsiella*	+	−	+	−
GG41E	Guigang	root	*Klebsiella*	−	+	+	−
GG42E	Guigang	root	*Klebsiella*	−	+	+	−
GG49E	Guigang	root	*Enterobacter*	+	+	+	−
GG50E	Guigang	root	*Klebsiella*	−	+	−	−
GG53E	Guigang	root	*Klebsiella*	−	+	+	−
GG160E	Guigang	root	*Klebsiella*	+	+	+	−
GG165E	Guigang	root	*Enterobacter*	+	+	−	−
LC89E	Liucheng	root	*Klebsiella*	+	+	−	−
LZ83E	Liuzhou	root	*Klebsiella*	+	+	−	−
LZ84E	Liuzhou	root	*Enterobacter*	+	+	+	−
LZ87E	Liuzhou	root	*Klebsiella*	+	+	−	−
LA3E	Longan	root	*Klebsiella*	+	+	+	−
LA4E	Longan	root	*Enterobacter*	+	+	−	−
LA11E	Longan	root	*Klebsiella*	−	+	+	−
LA14E	Longan	root	*Enterobacter*	−	+	−	−
NN143E	Nanning	root	*Enterobacter*	−	+	+	−
NN144E	Nanning	root	*Enterobacter*	−	+	+	−
NN208E	Nanning	root	*Enterobacter*	−	+	+	−
PG122E	Pingguo	root	*Klebsiella*	+	+	+	−
QZ80E	Qinzhou	root	*Enterobacter*	+	+	−	−
SS107E	Shangsi	root	*Klebsiella*	+	+	+	−
SS82E	Shangsi	root	*Klebsiella*	−	+	−	−

“+” presents positive, “−” presents negative
