# Supplementary material for: Plant Growth-Promoting Nitrogen-Fixing Enterobacteria Are in Association with Sugarcane Plants Growing in Guangxi, China
Source: Microbes Environ. 2012 Apr 18;27(4):391–8. doi: 10.1264/jsme2.ME11275 (PMC4103546; doi:10.1264/jsme2.ME11275)
Supplement: Supplementary file 1 [file 27_391_s1.pdf]

Table S1. Accession numbers for partial *nifH*, *anfH*, and *rrs* sequences of nitrogen-fixing bacterial isolates associated with sugarcane cultivar ROC22 grown in Guangxi

Isolate name	Genus affiliation	Accession no. for partial *nifH* sequences	Accession no. for partial *anfH* sequences	Accession no. for partial *rrs* sequences
CZ150S	*Klebsiella*	HQ204222		HQ204277
CZ152S	*Burkholderia*	HQ204223		HQ204278
CZ186S	*Klebsiella*	HQ204224		HQ204279
GG164S	*Klebsiella*	HQ204225		HQ204280
HX148S	*Enterobacter*	HQ204226		HQ204281
HX149S	*Klebsiella*	HQ204227		HQ204282
LA16S	*Klebsiella*	HQ204228		HQ204283
LC55S	*Klebsiella*	HQ204229		HQ204284
NN145S	*Enterobacter*	HQ204230		HQ204285
NN169S	*Klebsiella*	HQ204231		HQ204286
PG132S	*Enterobacter*	HQ204232		HQ204287
QZ25S	*Enterobacter*	HQ204233		HQ204288
QZ33S	*Klebsiella*	HQ204234		HQ204289
TD153S	*Klebsiella*	HQ204235		HQ204290
YL34S	*Enterobacter*	HQ204236	HQ204265	HQ204291
YX115S	*Klebsiella*	HQ204237		HQ204292
YX116S	*Klebsiella*	HQ204238		HQ204293
YX117S	*Klebsiella*	HQ204239		HQ204294
YX118S	*Klebsiella*	HQ204240		HQ204295
DX120E	*Klebsiella*	HQ204241		HQ204296
DX194E	*Klebsiella*	HQ204242		HQ204297
GG41E	*Klebsiella*	HQ204243	HQ204266	HQ204298
GG42E	*Klebsiella*	HQ204244	HQ204267	HQ204299
GG49E	*Enterobacter*	HQ204245		HQ204300
GG50E	*Klebsiella*	HQ204246	HQ204268	HQ204301
GG53E	*Klebsiella*	HQ204247	HQ204269	HQ204302
GG160E	*Klebsiella*	HQ204248		HQ204303
GG165E	*Enterobacter*	HQ204249	HQ204270	HQ204304
LC89E	*Klebsiella*	HQ204250		HQ204305
LZ83E	*Klebsiella*	HQ204251	HQ204271	HQ204306
LZ84E	*Enterobacter*	HQ204252	HQ204272	HQ204307
LZ87E	*Klebsiella*	HQ204253		HQ204308
LA3E	*Klebsiella*	HQ204254		HQ204309
LA4E	*Enterobacter*	HQ204255	HQ204273	HQ204310
LA11E	*Klebsiella*	HQ204256	HQ204274	HQ204311
LA14E	*Enterobacter*	HQ204257		HQ204312
NN143E	*Enterobacter*	HQ204258		HQ204313
NN144E	*Enterobacter*	HQ204259		HQ204314
NN208E	*Enterobacter*	HQ204260		HQ204315
PG122E	*Klebsiella*	HQ204261		HQ204316
QZ80E	*Enterobacter*	HQ204262	HQ204275	HQ204317
SS107E	*Klebsiella*	HQ204263		HQ204318
SS82E	*Klebsiella*	HQ204264	HQ204276	HQ204319

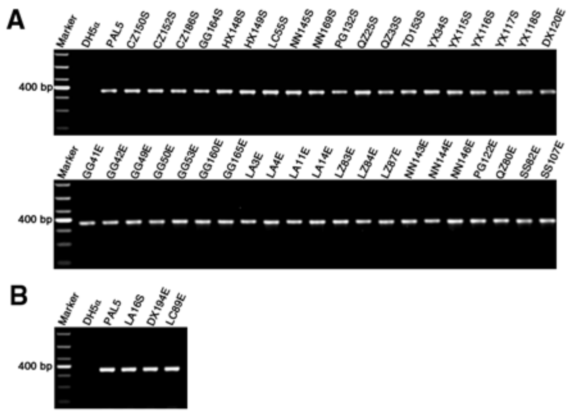

Fig. S1. 2% agarose gel electrophoresis for products of *nifH* PCR based on Z-primers from colonies of 40 ARA-positive isolates (**A**) and from colonies of isolate LA16S, DX194E, and LC89E (**B**). *E. coli* DH5α and *G. diazotrophicus* PAL5 were respectively used as negative and positive control.
